# A Review on Architectures and Communications Technologies for Wearable Health-Monitoring Systems

**DOI:** 10.3390/s121013907

**Published:** 2012-10-16

**Authors:** Víctor Custodio, Francisco J. Herrera, Gregorio López, José Ignacio Moreno

**Affiliations:** Telematics Engineering Department, Carlos III University of Madrid, Avda. Universidad 30, 28911 Leganés, Madrid, Spain; E-Mails: franciscojose.herrera.luque@uc3m.es (F.J.H.); gregorio.lopez@uc3m.es (G.L.); joseignacio.moreno@uc3m.es (J.I.M.)

**Keywords:** Wearable Health Monitoring Systems (WHMS), Body Area Networks (BAN), Wireless Sensor Networks (WSN), ubiquitous health, pervasive health, e-Textile, indoor location

## Abstract

Nowadays society is demanding more and more smart healthcare services that allow monitoring patient status in a non-invasive way, anywhere and anytime. Thus, healthcare applications are currently facing important challenges guided by the u-health (ubiquitous health) and p-health (pervasive health) paradigms. New emerging technologies can be combined with other widely deployed ones to develop such next-generation healthcare systems. The main objective of this paper is to review and provide more details on the work presented in “LOBIN: E-Textile and Wireless-Sensor-Network-Based Platform for Healthcare Monitoring in Future Hospital Environments”, published in the *IEEE Transactions on Information Technology in Biomedicine*, as well as to extend and update the comparison with other similar systems. As a result, the paper discusses the main advantages and disadvantages of using different architectures and communications technologies to develop wearable systems for pervasive healthcare applications.

## 1. Introduction

The demand for smart healthcare services, which allow monitoring patients' health status in a pervasive and noninvasive manner, is increasing as a means to alleviate the issues associated with costly welfare systems and an increasing elderly population, as well as to improve quality of life, bringing benefits to patients, medical personnel, and society [1,2].

As a result, outstanding research and development efforts have been carried out during the last years both by academia and industry in this area, driving great breakthroughs on enabler technologies, such as wireless communications, micro- and even nano-electronics, or sensing techniques and materials.

Advances in microelectronics and wireless communications have made Wireless Sensor Networks (WSNs) and Body Area Networks (BANs), which represent two key functional components in smart healthcare systems, a reality, despite the fact they present different features.

WSNs can be used to deploy low-cost and low-consumption communications infrastructure to support wide coverage and mobility (*i.e.*, pervasiveness), enabling natural movement of patients as well as potential development of value- added services (e.g., Location Based Services—LBS).

BANs are composed of tiny smart sensors deployed in, on, or around a human body [3]. Such sensors are deployed inside the human body in the so-called *In-Vivo* or Implantable BANs (IBANs). This kind of networks is still in an early development stage. Currently, they are mainly based on very specific wireless communications technologies, such as MICS [4]. In the long-term, they may be based on nanonetworks, a completely novel communications paradigm that aims at copying natural molecular and cell communications [5,6]. These sensors are distributed on the human body, measuring different physiological parameters, in the so-called Wearable BANs (WBANs), which represent the most widely used solution within this scope nowadays. Smart fabrics that combine conductive materials with organic textiles (e-textiles) are of special interest to the so-called WBANs, since they provide a comfortable and user-friendly way to monitor patient's health status over extended periods of time, avoiding the use of cables wired around the patient.

This paper is mainly focused on architectures and communications technologies for smart healthcare systems based on WBAN (so-called Wearable Healthcare Monitoring Systems—WHMS).

The remainder of the paper is structured as follows: Section 2 elaborates on typical architectures, features, and available communications technologies for wearable healthcare systems: In addition, Section 2 comprehensively surveys related work in this area. Section 3 uses the LOBIN platform to illustrate how e-textiles and WSN can be combined to develop smart healthcare systems providing not only real-time physiological monitoring but also additional services such as indoor location. Section 3 also compares the platform developed and the results obtained in the context of the LOBIN project with the related work presented in previous section. Section 4 discusses the most appropriate design criteria and decision making depending on the specific requirements of the target applications. Finally, Section 5 summarizes the paper and draws conclusions.

## 2. State of the Art

Smart WHMS definitely represent a very hot research topic, due to the fact that they may play a crucial role in present and future society. As a result, many research projects and prototypes have been developed during the last years and major breakthroughs in enabler technologies have been achieved in order to meet the specific requirements of these systems, as well as to face the challenges they present [3,7–15].

This section first discusses the typical communications architectures (based on a reference one) and the most relevant communications technologies used in this kind of systems. Next, a comprehensive survey on research projects, prototypes, and systems developed during the last years in this area is presented.

### 2.1. Architectures and Communications Technologies for WHMS

**Figure 1.**
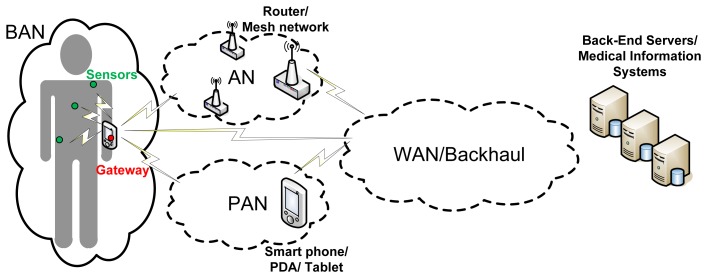
General Communications Architecture for WHMS.

Figure 1 shows a complete architecture for WHMS that fits the different approaches in [8–11]. However, wearable healthcare systems are so diverse that they will implement different combinations of such communications segments depending on the target application. In an application aiming at allowing the user or patient to monitor its health status on its own by using a smart portable device, such as a smartphone or a tablet, the Backhaul/WAN communications segment may not be needed; the sensors in charge of measuring the appropriate physiological parameters could communicate directly with the smart device making a BAN up. In case the raw data coming from the BAN is not processed locally at the smart device but at the “cloud” (e.g., to save battery in the smart device), the smart device would have to communicate with the back-end servers through the backhaul communications segment, so the communications architecture would be composed of the BAN and the Backhaul/WAN. In telemedicine applications aiming at remote diagnosis or remotely monitoring the health status of a patient or group of patients being at home, the backhaul network would be definitely needed and the information coming from the BAN may have to traverse an AN before reaching the backhaul segment of the communications architecture. If such application also takes into account information coming from devices or just things (as in the so-called Internet of Things) around the users, the gateway of the BAN and the objects around the user might form a PAN directly connected to the WAN and so to the medical information systems.

The communications technologies to be used will tightly depend on the specific requirements of each communications segment. For the WAN/Backhaul segment, cellular technologies (e.g., GSM, GPRS, UMTS) as well as broadband wired technologies (e.g., ADSL, cable) are the most appropriate, the first ones being the most widely used in practice, as it will be shown later in this section and in Section 2.2. Wi-Fi (IEEE 802.11) seems to be the most appropriate candidate for the AN. However, a wireless mesh network based on IEEE 802.11 or on IEEE 802.15—e.g., to provide coverage in indoor environments such as a hospital—that enables the communications with an eventual back-end system through a gateway, is also considered as an AN.

As it will be also illustrated later in this section and when reviewing the related projects in Section 2.2, Bluetooth is one of the most widely used communications technologies for PAN, since it provides reasonably high bandwidth (able to aggregate the traffic coming from the BAN) and it is widely implemented in commercial devices, such as smartphones or laptops. However, other communications technologies, such as IEEE 802.15.4/Zigbee or RFID, or even a combination of all them, can be also used to effectively deploy PANs.

Although many wired solutions are used to implement BAN in practice, as it will be shown at the end of this section and in Section 2.2, Wireless BANs (WBANs) are of special interest to this work since they meet better some key requirements of wearable healthcare systems, such as wearability, noninvasiveness, and comfortability. However, WBAN also need to overcome some issues related to reliability or security. Therefore, much research is currently being carried out to identify challenges at every layer of the communications stack and to propose solutions [9,12–15]. As a matter of fact, the IEEE 802.15 working group has recently launched the IEEE 802.15.6 standard exclusively targeting WBAN [16].

Table 1 summarizes and compares the most relevant communications for WBAN (both for Implantable—IWBAN and Wearable—WWBAN). It is worthwhile to remark that Bluetooth 4.0 Low Energy, despite not having been used in this kind of applications yet, represents a good candidate for WBANs, coupling its suitable technical features with its estimated high commercial penetration.

**Figure 2.**
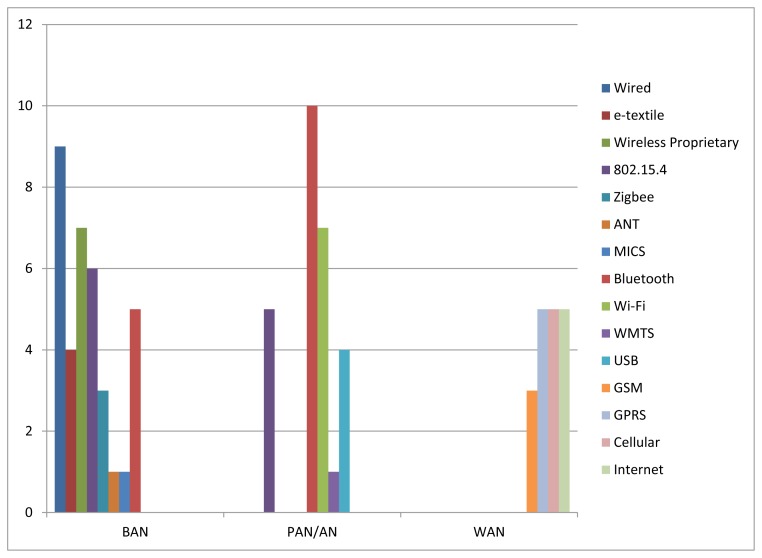
Communications Technologies and Communications Segments.

As a kind of conclusion and in order to illustrate current status on which are the most deployed communications technologies in each communications segment, Figure 2 shows the number of projects (from the subset of projects surveyed in Section 2.2) which use each communications technology in each communications segment.

As for the BAN, it can be seen that nine projects use wired solutions and four projects use e-textiles (which is a kind of wired solution as well) to communicate the sensors deployed around the body and the gateway of the BAN, meaning that 36% of the surveyed projects use some kind of wired solution in the BAN. IEEE 802.15.4/Zigbee represents the most widely used wireless communications technology in the BAN. There are six projects that work directly on top of IEEE 802.15.4 and three projects that use Zigbee (which in turn relies on IEEE 802.15.4), what represents 25% of the surveyed projects. Proprietary RF solutions are also widely used in the BAN (seven projects, representing about 20% of the considered projects). Finally, MICS and ANT represent two communications technologies that, despite not having been so used for the time being, will play a key role in IBAN and in BAN for sport applications respectively.

In the PAN/AN, wireless solutions are preferred instead of wired ones. Bluetooth is the most relevant communications technology for PAN. There are 10 projects which use Bluetooth in this segment. IEEE 802.15.4 is also widely used not only in PAN but also in AN, forming mesh networks which allow covering wide areas at low cost (there are five projects that use IEEE 802.15.4 as PAN/AN technology). Finally, Wi-Fi is the most widely deployed communications technology for AN, with seven projects using it in this segment.

Regarding WAN, Figure 2 shows that cellular technologies are the most widely used technologies in this segment. There are 13 projects that deploy such technologies (three projects use GSM, five projects use GPRS, and five projects state using cellular technologies, including GSM, GPRS and UMTS); whereas only five projects use regular Internet connections (e.g., ADSL, cable). Nevertheless, this choice tightly depends on the specific requirements of the target application (e.g., mobility, pervasiveness, ubiquity). For example, in an application aiming at monitoring the health status of firefighters in the field, cellular technologies will be definitely used. However, in a telemedicine application aiming at monitoring health status of patients while they are at home, regular Internet communications technologies may be the best option. Next, the most relevant projects in this area that have paid special attention to the communications perspective are analyzed in detail.

### 2.2. Related Work

During the last years, several research projects on WHMS have been developed focusing on different medical areas. In this paper, projects in which the design of the communications architecture is part of the focus of the research activities have been selected. They are presented in chronological order and compared in Table 2. Subsequently, more detailed project descriptions are provided by means of individual tables.

In Table 2, systems are first classified according to the “Medical Area” they are designed for into broad sense categories. Specific target application can be found in the individual project descriptions. This classification criterion must not be used to get an impression of the medical field interest of the researchers on WHMS, since only those projects elaborating on the architecture and communications technologies have been considered. It is worthwhile to mention that projects belonging to the General Purpose and Cardiac categories have in common the monitoring of heart related physiological parameters. The distinction is made because projects assigned to the latter category are focused on such organ and only track heart related parameters while those assigned to the former also consider other kind of medical parameters to evaluate the overall health status.

As previously stated in Section 1, an essential feature of WHMS is pervasiveness. Such a feature can appear in different flavors depending on the specific target application, justifying the classification criterion denominated as “Ubiquity Level”. According to it, three categories can be distinguished, namely “Controlled Area”, “Wide Area” and “Self-Monitoring”. “Controlled Area” and “Wide Area” systems have in common the presence of medical specialists playing the role of information users. The former type includes those systems that allow for patients monitoring inside the boundaries of a specific area, usually a hospital facility, as in LOBIN [44]. The latter refers to those systems where patients can virtually have any arbitrary location (inside the coverage area of WAN technology, typically a Cellular Network technology). In “Self-Monitoring” systems patients can also have any arbitrary location but there is no involvement of medical specialists. In this case, patients are provided feedback on its own medical status. Some systems provide information to both medical specialists and the patient while not imposing limits on patient's location. Such systems are assigned to both the “Wide Area” and the “Self-Monitoring” categories.

From the communications point of view, systems can be compared in terms of the network segments that make up their architectures and the communication technologies used in each of them. In this sense, selected projects are analyzed taking as reference model the General Communications Architecture for WHMS introduced in Section 2.1 (Figure 1). Abbreviations are used to specify the concrete communications technology used in the network segments that are deployed in each system. For non-deployed network segments corresponding fields are left blank.

More detailed descriptions of selected projects follow. They are done in terms of individual tables specifying the same concepts for each system, so that the different systems can be further compared. Such concepts have been chosen with the purpose of providing the reader with a general overview of both the medical purpose and the architecture of the system, and include:

- “Project name/Funding”: whenever defined, the project name is used to refer to the system, otherwise paper authors are used. Funding can be provided either by specific research funding programs or by research institutions. System presentation year is also specified.
- “Target Application”: states the specific medical purpose of the system.
- “Tracked Parameters”: lists the medical parameters that the system considers classified into two different categories, namely physiological and non-physiological. For those parameters that appear in more than one system, abbreviations are used. Such parameters are:
  □ “Physiological” parameters: Blood Pressure (BPr), Electrocardiogram (ECG), Electroencephalogram (EEG), Electromyogram (EMG), Galvanic Skin Response (GSR), Heart Rate (HR), Photoplethysmogram, Respiration (R), Blood Oxygen Saturation (SpO2) and Temperature (T;.
  □ “Non-physiological parameters”: Activity (A), Acceleration (Acc), Humidity, Location (L), Movement (M), Position (P;, Voice and Weight.
- “System Architecture”: provides a brief description of the main building blocks composing the system considering both hardware and software elements.
- “Communications Technologies”: states the concrete communications technologies used in each network segment of the system (“BAN”, “PAN/AN” and “WAN/Backhaul”), taking as a reference the General Communications Architecture for WHMS presented in Figure 1. In case the system does not implement any of the network segments the corresponding field is left blank.
- “System Highlights”: in this field, peculiar features of the system are commented.

System descriptions are presented in chronological order below from Tables 3–38:

## 3. The LOBIN Prototype: A Practical Study Case

### 3.1. System Description

The LOBIN system is defined as a healthcare IT platform to both monitor several physiological parameters (ECG, HR, angle of inclination, activity index and body temperature) and track the location of a group of patients within hospital facilities. The system shows and stores the data associated with the patients in real time. The device used to measure the physiological data is wearable, non-invasive, comfortable, and washable. The location algorithm used is accurate enough to determine correctly the hospital room where a given patient is located. Furthermore, the system supports the configuration of alarms by setting different triggers tailored to each patient and the transmission of certain parameters when the Management System explicitly requests them (on-demand) or after any alarm occurs.

**Figure 3.**
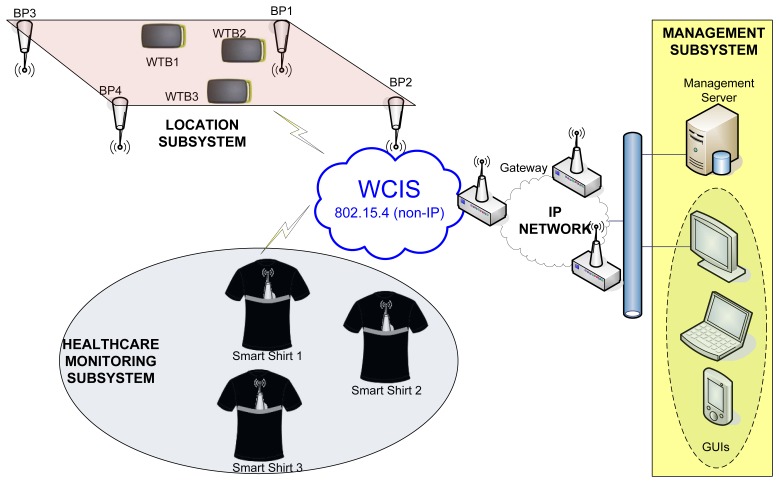
System Architecture.

Figure 3 shows the overall architecture of the LOBIN system. Such architecture is composed of the following subsystems:

- The Healthcare-Monitoring Subsystem consists of a set of smart shirts to be worn by the patients. Every smart shirt is equipped with a device (so-called Wearable Data Acquisition Device—WDAD), which collects and processes the physiological parameters and transmits them wirelessly.
- The Location Subsystem consists of a set of Beacon Points (BPs), which are deployed in well-known positions, and a set of end devices (so-called Location Wireless Transmission Boards—LWTBs), which are carried by targeted users (e.g., patients or any other personnel from the hospital). The BPs, as their name suggests, send beacons periodically with well-known transmission power. The LWTBs collect signal strength information received from different BPs and send it wirelessly.
- The Wireless Communications Infrastructure Subsystem (WCIS) is placed in between the Location and Healthcare-Monitoring Subsystems and the Management Subsystem. It is responsible for carrying data from the former to the latter and commands from the latter to the former. It consists of a set of devices (so-called Distribution Points—DPs) that transmit ad hoc data up to a Gateway, which forwards them to the Management Subsystem. Thus, the Gateway interfaces with the WCIS (non-IP-based) and with the wired communications infrastructure (IP-based) connected to the Management Subsystem.
- The Management Subsystem represents the Information Technology (IT) infrastructure that handles the information associated with every single patient. It consists of a Management Server, which processes and stores all the data associated with the patients, and a Graphical User Interface (GUI), which allows the hospital staff to monitor the status of the patients. This subsystem can be integrated into commercial hospital management systems.

Next, each subsystem is explained in detail.

#### 3.1.1. Healthcare Monitoring Subsystem

The Healthcare Monitoring Subsystem represents the BAN of the LOBIN project and it is mainly based on smart fabrics and e-textile. Thus, the LOBIN BAN can be classified as a wired solution from the communications point of view. Other surveyed WHMS have opted for wireless solutions, such as Bluetooth and IEEE 802.15.4 (e.g., Jovanov *et al.* [26,27], Zhang *et al.* [49], and O'Donovan *et al.* [43]). There are also WHMS that use proprietary technologies, such as Fensli *et al.* [20], which uses RF-transmitter at 869.700 MHz, or Human++ [34], which uses RF 2.4 GHz ISM. Other WHMS work with specific technologies, such as MICS (e.g., Yuce *et al.* [45]) in the medical field or ANT (HipGuard [39]) in the sports sector.

**Figure 4.**
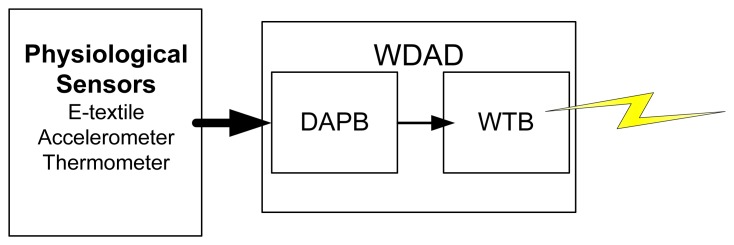
Healthcare Monitoring Subsystem Block Diagram.

The Healthcare Monitoring Subsystem consists of the set of smart shirts to be worn by the patients. Every smart shirt is equipped with physiological sensors and a WDAD, which processes the data coming from the sensors and transmits them wirelessly. The WDAD is further divided into two different PCBs: the Data Acquisition and Processing Board (DAPB) and the WTB. Figure 4 sketches how the Healthcare Monitoring Subsystem works.

**Figure 5.**
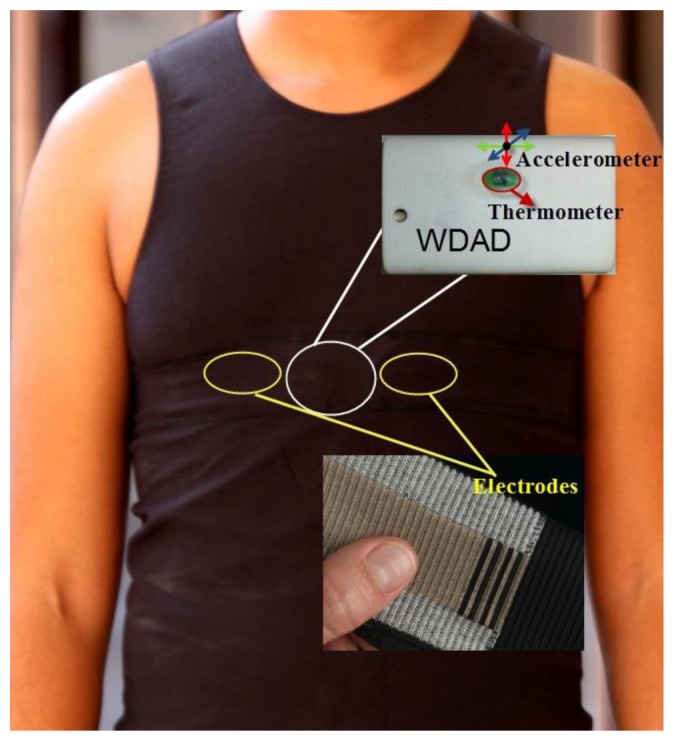
Physiological Sensors.

The physiological sensors are in charge of measuring raw data that will be further processed in order to obtain the required biomedical parameters. The available sensors are: the e-textile electrodes, the accelerometer, and the thermometer. The e-textile electrodes are used to measure the bioelectric potential of the human body and are integrated into the smart shirt, as shown in Figure 5. The signals provided by the 3-axis accelerometer are used to detect patient movements and determine whether the patient is laying down or moving about in order to aid appropriate diagnosis. The thermometer measures the body temperature and it must be in direct contact with the skin of the patient. Both the 3-axis accelerometer and the thermometer are integrated into the WDAD, as it is also shown in Figure 5.

The DAPB collects all the data from the sensors, processes them, merges them all together in a message (*i.e.*, the healthcare-monitoring subsystem frame) and sends them via a serial port to the WTB.

**Figure 6.**
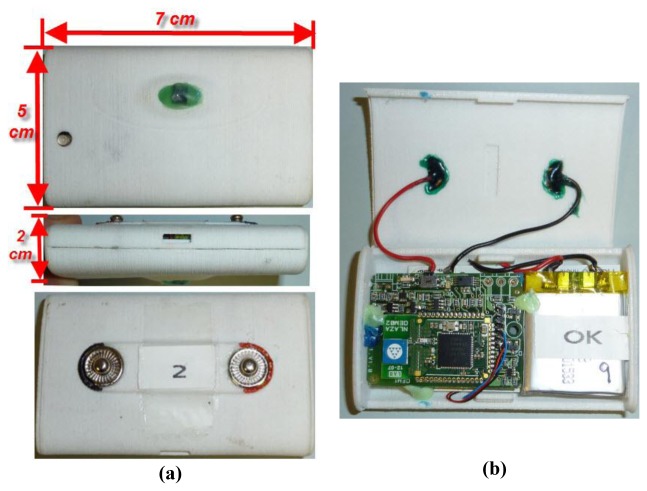
(**a**) Wearable Data Acquisition Device (WDAD) (**b**) Healthcare Monitoring Wireless Transmission Board (WTB).

The WTB builds a new packet by adding information related to the WCI to the message coming from the DAPB and transmits it wirelessly. Both the DAPB and the WTB share the same battery so they can be integrated into a common PCB for commercialization. Figure 6 shows the developed hardware.

As has already been mentioned, a healthcare-monitoring frame to pack all the sensitive information in just one message is defined. All the physiological parameters are sampled every 4 ms. However, this message is only transmitted after collecting 65 ECG samples. This value is determined by the size of the frame resulting from the healthcare-monitoring frame together with the additional routing information, needing to be as close as possible to the 802.15.4 MTU (102 bytes). As a result of this decision, efficiency is maximized and transmission rates decreased, which in turn reduces collisions in the WCI. Thus, from the Management Subsystem point of view, the rest of the parameters apart from the ECG are sampled every 260 ms. Table 39 summarizes the most important features of the parameters transmitted in this message.

Among the cardiac monitoring systems studied, the key monitored parameter is the ECG. Many projects, such as RECAD [21], C. Park *et al.* [32], MASN [38], or GeM-REM [52], are based only on the monitoring of such parameter. Other projects, like LOBIN, monitor additional parameters, such as temperature, activity index and the relative position of the body, which can help caregivers to understand the changes in the ECG of the patient. Not only these parameters are used to complement the ECG; the respiration (monitored in WEALTHY [24] and Magic [23]), the blood pressure (monitored in Chung *et al.* [37] and Personal Health Monitor [35]), and the SpO2 (monitored in Jovanov *et al.* [26,27] and LifeGuard [22]) are other important physiological parameters used to do so.

There are also different approaches with regard to the processing of the collected information. While in LOBIN all information is sent, shown and stored in real time, there are other projects that, because of communications limitations, only send information periodically or on demand (e.g., AMON [19]), or that, in order to save energy, only send the information when any abnormal behavior is detected (e.g., GeM-REM [52]). In addition, there are some projects that combine monitoring with artificial intelligence to analyze and diagnose cardiovascular issues (e.g., Shengh *et al.* [47]).

#### 3.1.2. Location Subsystem

Some of the surveyed projects do not have tracking systems, either because the user location is always known or because it is not necessary. However, there are some other projects, including LOBIN, in which it is useful to be able to accurately locate patients and caregivers, since, by knowing their exact positions, the system can quickly lead the closers medical professionals to the desired locations, thus saving treatment time and avoiding major problems. Within these projects, there are some which perform outdoor location, such as MIThril LiveNet [17,18], Jovanov *et al.* [26,27], SmartVest [33], Personal Health Monitor [35], HeartToGo [40], and KNOWME [54]; whereas others use indoor localization, such as LOBIN [44], CodeBlue [25], and MASN [38].

In the case of outdoor localization, the use of GPS is unanimous, although there are others different methods. As for the indoor localization, much research has been carried out recently and many different solutions using different technologies, such as Assisted-GPS (AGPS) [55], 802.11 [56], Bluetooth [57–59], RFID [60,61], Ultra Wide Band (UWB) [62], or IEEE 802.15.4/Zigbee [63–65], have been explored. The use of each technology implies some benefits and drawbacks that make it more or less suitable depending on the targeted scenario. In this case, the three aforementioned projects use IEEE 802.15.4, since it represents a good solution for sensor networks that run on limited battery power.

**Figure 7.**
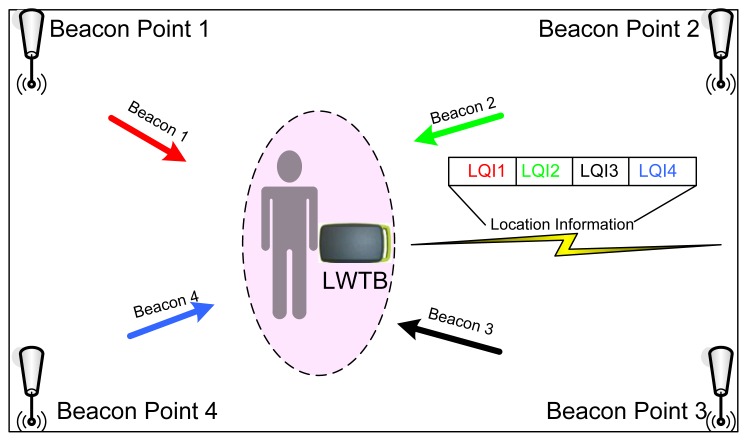
Overall Location Subsystem architecture.

While CodeBlue [25] and MASN [38] use an empirical localization scheme, the so-called MoteTrack, which matches the radio signature acquired by a roaming device with known locations by using a database that stores average radio signatures and know locations, LOBIN uses an indoor location algorithm based on Weighted Centroid Localization (WCL) and the LQI. The LQI measurement is a characterization of the strength and/or quality of a received packet [63–65]. The WCL algorithm [66] consists of computing a point comprised within the area covered by the BPs the target received beacons from, so that this point will be closer to those BPs from where higher LQIs are received. In order to do so, different weights (directly proportional to the received LQI) are assigned to every received beacon. This solution was chosen among the many different available ones since it represents a nice trade-off between accuracy and battery consumption. As it has already been mentioned, LOBIN Location Subsystem consists of two different devices: BPs and LWTBs. Figure 7 sketches how the Location Subsystem works.

The BPs are deployed in well-known positions and are plugged into the electricity supply network. They send beacons periodically in one of the four channels scheduled for location purposes (namely, IEEE 802.15.4 channels 11, 12, 13, and 14) using a fixed and well-known transmission power.

The LWTBs are carried by the users and are in sleep mode most of the time. Periodically (eventually, every 9 s, as explained in section Location Subsystem Tests), they wake up and listen to every single channel available for location purposes in order to record the LQI received from the different BPs. Note that the time that the LWTBs are listening to a given channel has to be at least twice the time between beacons from a given BP in order to avoid beacon losses. Once these data is collected, the LWTBs merge them into a single message (*i.e.*, the location subsystem frame), add some necessary network layer information and send it to the Management Subsystem through the WCI Subsystem.

The location subsystem frame consists of a set of pairs (BP identifier, LQI). This set is preceded by a field that identifies the number of transmitted pairs up to a maximum of 80, which is more than enough taking into account the areas to be covered by the system and the transmission range of the BPs. The location of the patients is computed at the Management Subsystem—using such pairs and the WCL algorithm—and then drawn onto a 2-D plan. It is worthwhile to remark upon the fact that the location algorithm needs at least four pairs (BP identifier, LQI) in order to work properly.

#### 3.1.3. Wireless Communications Infrastructure Subsystem

The LOBIN WCIS as well as other surveyed WHMS such as CodeBlue [25], MASN [38], Lee *et al.* [42], and MEDISN [46] have decided to use IEEE 802.15.4 in their ANs because it is a technology with low bandwidth, low power consumption and low deployment and maintenance costs. Others WHMS have opted for the choice of technologies more developed and widely deployed at the time, such as IEEE 802.11 (e.g., AUBADE [31], MIThril LiveNet [17,18] or SmartVest [33]).

**Figure 8.**
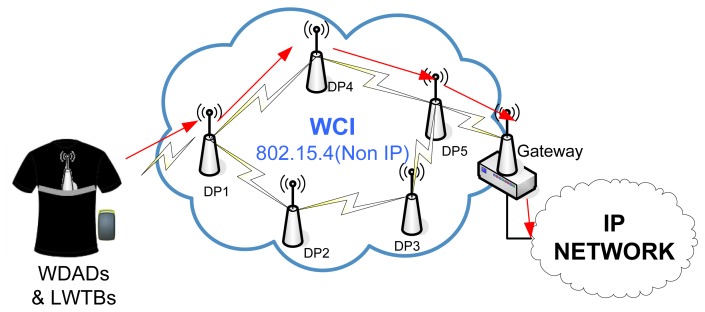
Overall WCI Subsystem architecture.

The WCIS consists of a network of DPs that transmit data—coming from the WDADs and the LWTBs—*ad-hoc* up to a Gateway, which forwards them to the Management Subsystem. The overall WCI Subsystem architecture is shown in Figure 8.

DPs are deployed all over the targeted areas and they are plugged to the electricity supply network. From the hardware point of view, they are exactly the same as the BPs, the only difference being the software that runs on them.

The Gateway incorporates a wireless interface and an Ethernet (IEEE 802.3) interface. They are in charge of forwarding all the data coming from the WCI to the Management Subsystem. They are plugged into the electricity supply network. They implement full routing functionality, exactly as DPs do. Moreover, WDADs and LWTBs can send data straight through them.

Regarding the routing algorithm used in the WCIS, over the last few years much research has been carried out in order to design and develop routing algorithms that fit the many restrictions of IEEE 802.15.4 networks in an effective way [67–70]. These restrictions have to do mainly with the limitations of the available hardware (e.g., battery, memory, computing capacity) and with some other issues associated with wireless communications, such as scarce bandwidth or collisions. The scenario considered in the LOBIN project adds some additional complexity to the problem, since it presents an asymmetric traffic pattern. Most of the information is sent from the patients to the Management Subsystem (uplink), since patients are sources of data; whereas only a few commands travel in the other direction, *i.e.*, from the Management Subsystem to the patients (downlink). As a result, a routing algorithm that fits such special features has been designed and developed on top of IEEE 802.15.4 [71].

The routing algorithm used in the WCI Subsystem is a source routing algorithm based on Dynamic Source Routing (DSR) [72]. When a DP is turned on, it queries its neighbour DPs to discover how to reach the destination (*i.e.*, the Gateway). Thereafter, it receives responses, which contain different paths to do so. The first received response is stored as the default route. The following responses (up to 2) are stored as back-up routes. Before storing such routes, it is checked that they do not contain the same DP several times in order to avoid loops. In addition, DPs exchange, periodically, status information with their neighbours. Nevertheless, since the targeted scenario is very stable, this period is set to a high value (namely, minutes) in order to reduce the overhead introduced into the WCI. If a DP realizes that the default path is down either while transmitting a packet or after exchanging routing information, it can use one of the other default paths to solve the problem immediately. If there is no back-up path available in the DP at any given moment, the path discovery procedure is triggered again [71].

In order to save memory in the DPs, they are not aware of the end nodes (namely, WDAD or LWTBs) that are associated with them, *i.e.*, they do not store such information in memory. However, end nodes do store the DP they are associated with. If, either because the end node roams from one DP to another or because the DP it is associated with goes down, the end node realizes its DP is no longer available and it looks for other DPs to send the data through. Furthermore, in order to save battery life, end nodes are not involved in routing [71].

In order to avoid possible interferences with the Location Subsystem, as well as with other widespread communication technologies such as IEEE 802.11 [73], the 802.15.4 channel 25 is used for communication within the WCIS, since it is the furthest one from the channels used to broadcast location beacons.

Thus, among the WHMS studied based on IEEE 802.15.4, two groups can be differentiated according to their network and application layer. On the one hand, there are some projects that work directly on top of IEEE 802.15.4, designing and developing their own network and application layers (e.g., LOBIN, MASN [38], that adopts a cluster-based communication scheme as its routing protocol, MEDISN [46], in which their distribution points are self-organized into a routing tree, and CodeBlue [25], which is based on a publish/subscribe routing framework allowing multiple sensor devices to relay data to all receivers). On the other hand, there are some projects that use Zigbee (in the body area network) such as Chen *et al.* [41], and O**'**Donovan *et al.* [43]). The network layer of Zigbee natively supports both star and tree topologies, as well as generic mesh networks.

#### 3.1.4. Management Subsystem

**Figure 9.**
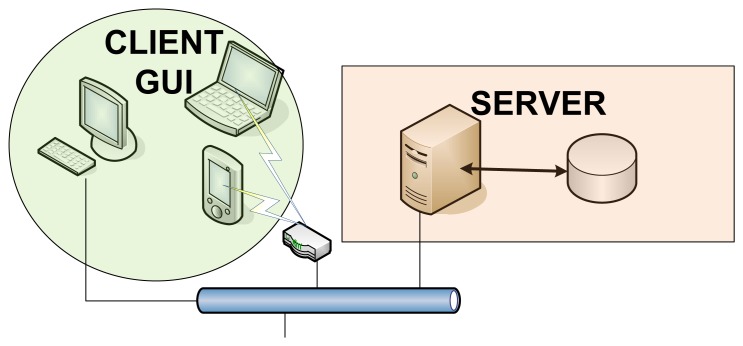
Management Subsystem client-server architecture.

The Management Subsystem is based on a client-server architecture (shown in Figure 9), the Management Server being the server and the GUIs being the clients.

The Management Server was developed using C as programming language. It runs on Fedora, which is an operating system built on top of the Linux kernel. It uses an Oracle database (DDBB) to store the information associated with the patients. The DDBB provides an independent interface with the GUI, thus allowing the development of tailored user applications to cover additional functionalities.

The GUI was developed in Java, which provides it with great flexibility and allows it to run on any platform without problems, a computer, a PDA or a mobile. The developed GUI meets the system requirements, since it allows managing patients' profiles, monitoring all the medical parameters of any patient in real-time, locating any patient within the hospital facilities, verifying if any alarm has been activated, as well as sending an SMS including this information if required.

The Management System also provides tools for managing maps and BPs' locations, so that this application can be implemented in any hospital without modifying the software of the location module.

### 3.2. System Validation and Tests

During the validation of the system, each subsystem was tested first separately for debugging and tuning purposes. Finally, the overall system was tested in a real scenario (namely, as a pilot scheme deployed in the Cardiology Unit of La Paz Hospital, Madrid, Spain).

#### 3.2.1. Healthcare Monitoring Subsystem Tests

The Healthcare Monitoring Subsystem tests were planned twofold. The main goal of the first subset of tests was to verify that the sensors collect real data and that the WDAD processes and transmits these data correctly. In order to verify the correct processing of the bioelectric potentials obtained by the e-textile electrodes, the values sent by the WDAD were compared with the real values generated in the laboratory using the generator NI USB 628 from National Instrument. To verify that the body temperature sensor works properly, its values measures were compared with the values of a commercial thermometer, concluding that the temperature taken by the thermometer integrated into the WDAD takes about 5 minutes to acquire an accurate value. The accelerometer tests were performed manually by tilting the device to known angles.

Next, the second subset of tests was devoted to verify that the sensors work properly when in direct contact with the human body. The main result from this subset of tests has to do with the quality of the acquired ECG signal. It was checked that the ECG signal quality is more than acceptable when patients stay still (*i.e.*, normal conditions), but worsens when sudden movements take place (e.g., when patients get out of bed or walk around). Nevertheless, in the latter situations, the measured ECG signal is much more accurate if the electrodes are wet or covered with a conductive gel. In addition, the ECG signal quality in such situations can be further enhanced by filtering the noise taking advantage of the signals provided by the 3-axis accelerometer [42].

#### 3.2.2. Location Subsystem Tests

The Location Subsystem tests were also divided into several stages. The first subset of tests was devoted to figure out what is the optimum gap between BPs. BPs are deployed following a rectangular grid in the hospital facilities ceil. The closer they are, the better the location algorithm performs, since it is most unlikely that a LWTB loses a beacon from a BP or that a LWTB receives beacons from less than four BPs, but the more expensive the deployment is, because more BPs are needed to cover the same area. Different configurations were tested, deploying BPs in rectangles of 100, 150, and 200 m^2^, the best results being obtained for the rectangles of smallest area. Therefore, our recommendation as a design criterion is that the area of the sub-rectangles of the BP grid network should not exceed 100 m^2^.

The main goal of the second subset of test was to maximize the performance of the Location Subsystem for the chosen DP deployment configuration by tuning different parameters. The parameters that were considered are:

- The period of time LWTBs send information (Ptx_loc)
- The period of time Beacon Points send beacons (Pbeacons)
- The time end nodes spend listening to each location channel from the 4 available ones (Tlisten)

Note that the lower Ptx_loc is selected, the more accurate the patients' locations drawn in the map are, since the Management Server receives location information more frequently, but the more battery LWTBs spend, because they send information more frequently too. Furthermore, it has to be taken into account that Ptx_loc must be always higher than the time the end node needs to listen to the 4 channels available for location and that Tlisten must be at least twice Pbeacons in order to ensure there is enough time to receive at least one beacon:

(1) Pix_loc≥Tlisten⋅Nchannels

(2) Tlisten≥2⋅Pbeacons

The five configurations shown in Table 40 were tested and it was checked qualitatively that the configuration set in test number 5 performed better than the others, so it is the recommended one.

In this configuration, Ptx_loc is set to the minimum value in order to reduce the latency and to improve the location of patients when they are moving around. To reduce the probability of losing a beacon, Tlisten is set to 4 times Pbeacons. Hence, if a beacon gets lost there is still enough time to receive at least another two. This decision improves significantly the stability of the location algorithm.

The error margin of the location algorithm was proved to be around 2 m^2^. This error margin improves the results obtained in [66], despite the fact that the results presented in [66] were obtained outdoors. This is mainly because the BPs are placed much closer to one another in this case than in the experiments carried out in [66] (10 m × 10 m in these tests and 43 m × 43 m in [66]). However, this accuracy is similar to the one reported in CodeBlue [25], where the location algorithm is based on RSSI signatures.

#### 3.2.3. WCIS Tests

**Figure 10.**
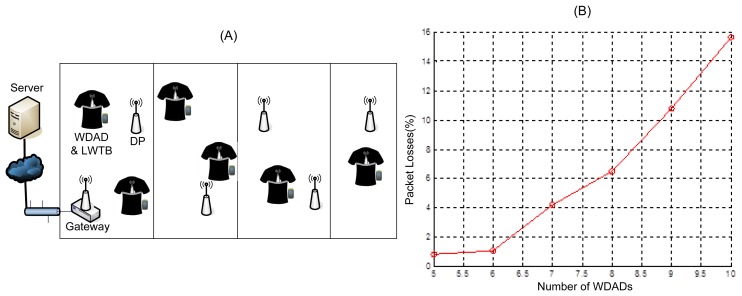
(**A**) Distribution Point network test (**B**) Test results for DP network topology.

The main goal of the WCI Subsystem tests was to determine the topology that suits better the target scenario. The first test deployment was composed of a network of DPs and a Gateway. In order to cover approximately 300 m^2^, five DPs and one Gateway were deployed, as Figure 10(A) shows. The main requirement the chosen topology has to meet is that the system supports at least five users ensuring that the percentage of lost packets is not more than 2% of those sent. It is worthwhile to remark upon the fact that these tests were run in a lab where intensive IEEE 802.11 tests were carried out in parallel. Thus, as it has been pointed out in Section 3.1.3, IEEE 802.15.4 channel 25 was selected in order to alleviate possible interference issues [73]. In addition, the WCIS was deployed in the roof of the lab, which is built of metal structures and the lab itself is plenty of metal structures, which may affect the propagation of the electromagnetic signal.

First, a scenario composed by a network of DPs and a Gateway was tested. Obtained results show that this topology does not support high rate applications. Figure 10(B) shows that the percentage of lost packets increases non-linearly with the number of patients and greatly exceeds the maximum loss percentage allowed. This is due to the fact that the traffic handled by the network does not grow linearly with the number of patients, but higher, since it not only increases the traffic added by the sources, but also the traffic forwarded by the DPs. Similar issues were also reported in [25].Therefore, this configuration is suitable for applications that present low traffic load, but require high coverage (e.g., ordinary temperature checking on-demand or location fixing), but it does not work for high rate applications (e.g., real-time ECG monitoring) since it does not support more than 6 WDADs sending messages every 260 ms (∼3.1 Kbps).

The same network topology (*i.e.*, DP network) duplicating the deployed infrastructure was also tested. In this scenario two different channels were used within the WCIS in order to split the traffic. Several pairs of IEEE 802.15.4 channels were tested and none of them yielded the expected improvement. Thus, it can be concluded that in such scenarios that present heavy traffic load, interference between IEEE 802.15.4 channels may occur with subsequent high data losses. Furthermore, this approach entails some drawbacks such as that LWTBs have to be programmed to work in one channel or another or that network infrastructure has to be duplicated, which means an increase on the deployment cost.

**Figure 11.**
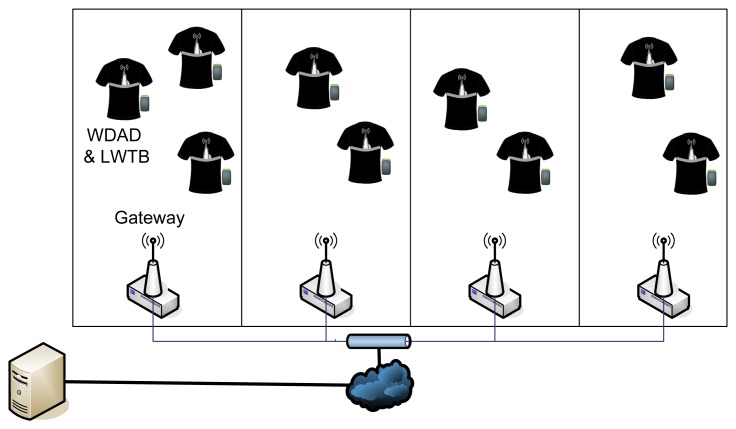
Gateway network test.

Finally, the DP network was replaced by a network of Gateways (Figure 11). This topology was proved to provide the best results, presenting the lowest percentage of losses in scenarios with 8/10 WDADs and LWTBs.

This percentage of losses was tested to be with a 95% confidence in the interval (0.2068%, 1.6585%) for 10 tests of about 15 minutes duration involving 10 users. Therefore, the obtained data were considered to fit a Gaussian distribution and the 95% confidence interval was computed using the formula below, μ being the mean of the data, σ the standard deviation of the data, and n the length of the data (*i.e.*, n = 100):

(3) $$\left(\mu -1.96\frac{\sigma }{\sqrt{n}},\mu +1.96\cdot \frac{\sigma }{\sqrt{n}}\right)$$

This approach adds redundancy to the system and supports user mobility better, since sources may be within the coverage of different Gateways and so data may be forwarded to the Management Server several times, reducing the probability of packet losses even when a patient is moving. Thus, the reliability of the system is improved and the user mobility is supported without interruption. However, this WCI configuration presents also some drawbacks, such as the wired infrastructure is required and the number of duplicated packets in the Management Server increases dramatically, although this is not a problem since the Management Server handles packets much faster than the rate they arrive with.

In others WHMS using IEEE 802.15.4 as communications technology, both stress and packet loss tests were also performed. In MEDISN [46], 3 patients sent monitoring information at the same time and the percentage of packets lost was of 0.65%. In MASN [38] the number of traffic sources was increased from 20 to 150, yielding a percentage of packets lost between 5% and 20%. In addition, the user mobility causes packet losses in most of the WHMS which support it. For instance, in MASN [38], several seconds (between 6 and 15 s) are lost from a source when it changes of cluster, and in MEDISN [46], the percentage of lost packets increases up to 3.3%.

#### 3.2.4. Overall System Tests

**Figure 12.**
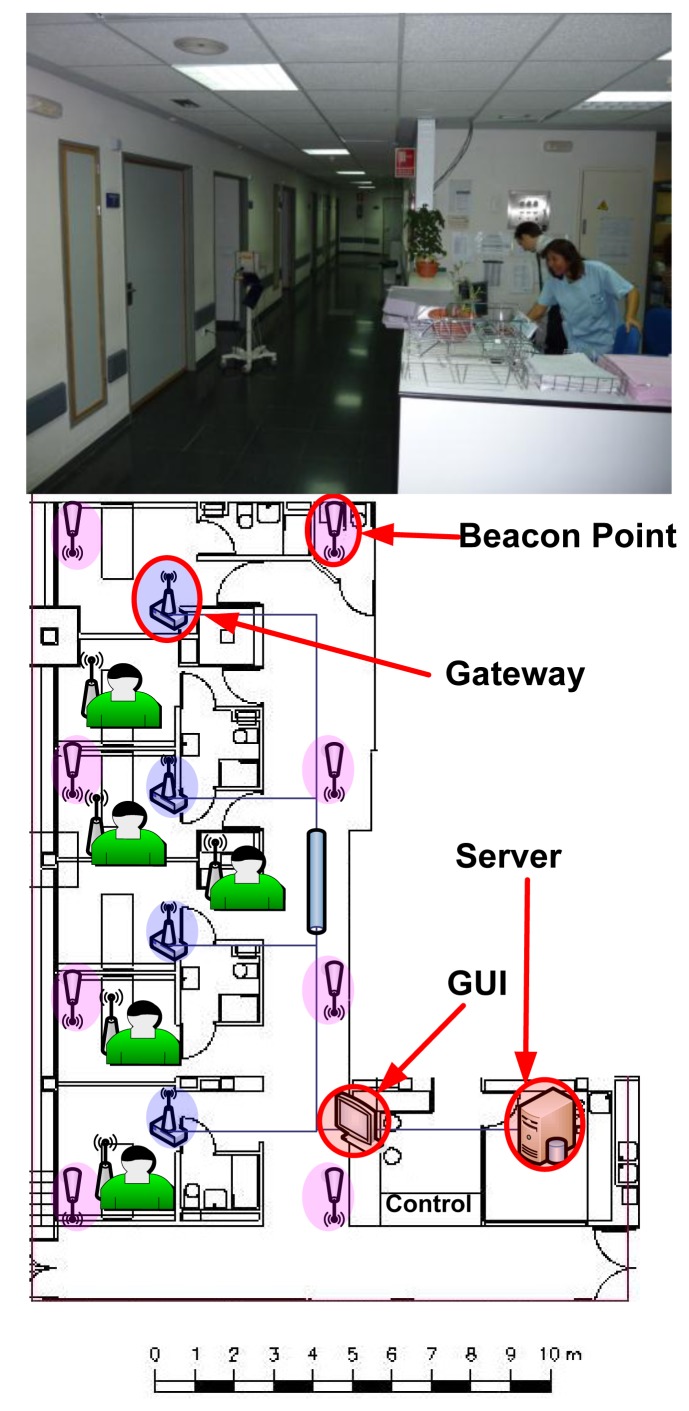
Pilot scheme schematics.

After debugging and tuning each subsystem separately, the performance of the whole system was tested in a real scenario by deploying a pilot scheme in the Cardiology Unit at La Paz Hospital (Madrid, Spain). For this client-side validation, each subsystem was set up following the design criteria and conclusions from previous tests. Figure 12 illustrates the coverage area and network infrastructure that was deployed for the pilot scheme.

The tests carried out at the hospital facilities involved five patients (*i.e.*, five WDADs and five LWTBs) and ran for 24 hours. Such tests were considered successful, since the results obtained results met the expected ones (and so the requirements of the system):

- Percentage of packet losses was lower than the maximum acceptable value (*i.e.*, 2%)
- Battery life was proved to be about 8–9 hours for WDADs and two days for LWTBs
- The location algorithm was tested to work properly
- Measured physiological parameters were normal and met expected values
- The quality of the real-time ECG signals was approved by hospital doctors

In addition, hospital personnel provided valuable feedback to improve the quality and usability of the system, such as:

- The tightness of the smart shirt was pointed out as an issue by hospital personnel, since patients from Cardiology Unit often present high abdominal perimeter. Nevertheless, no further complains were received.
- Hospital staff use the GUI both to monitor the patients' status in real-time easily, sitting in front of a PC, and to look at the signals over a long period of time, taking advantage of the features provided by the tool. Some of these features, such as checking just a given period of time by selecting initial time and final time or the scroll, were incorporated into the GUI on the request of hospital personnel.
- The ECG display was modified following the feedback from the hospital personnel in order to fit the ECG representation they are used to handle (e.g., from commercial electrocardiographic devices).

**Figure 13.**
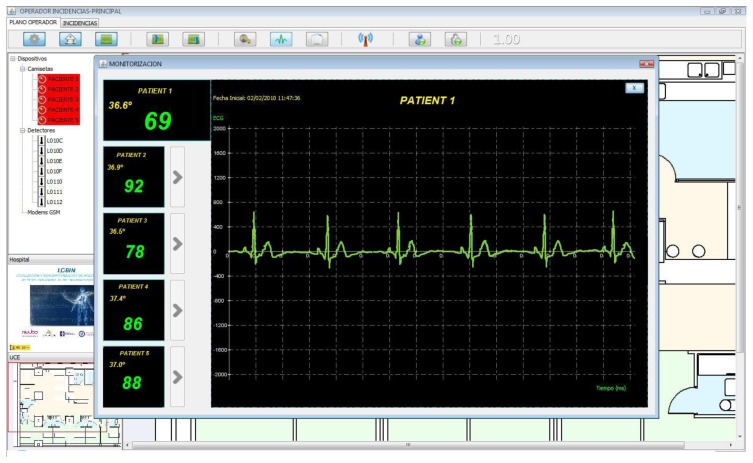
Real-time ECG.

Figure 13 shows the developed GUI, where the health status of five patients can be monitored at a glance and the ECG of one of them is displayed. The tool also includes a Geographic Information System (GIS) to locate the patients in the hospital facilities plan.

## 4. Discussion

In the field of WHMS the design of the communications infrastructure is a critical point. An extensive study on such systems has been done for the preparation of this paper in which different communications architectural solutions and communications technologies have been analyzed. As a result some basic WHMS design guidelines can be drawn.

Regarding the communications architecture, as it has been previously shown it can be divided into different network segments, namely BAN, PAN/AN and WAN/Backhaul (Figure 1). Despite every system implements its own solution in this sense, they all have in common the presence of at least a BAN segment. According to this and depending on the concrete “Ubiquity level”, advices on the additional necessary network segments can be given:

- “Self-Monitoring” systems may probably require a PAN network segment to provide connectivity with a data processing device that is not part of the BAN, as in LifeGuard [22]. Despite this such segment might not be necessary, like in HealthGear [28].
- “Controlled Area” type systems communications architecture may also need the addition of a PAN network segment to the BAN segment in order to expand the coverage area. An example of this is MASN [38]. Again this segment might not be necessary, as in Nerino *et al.* [50].
- “Wide Area” systems compulsory require a WAN/Backhaul network segment to allow for the remote monitoring. All the analyzed systems belonging to this category implement it.

From the communications architecture point of view, systems which ubiquity level corresponds to both the “Wide Area” and the “Self Monitoring” are equivalent to “Wide Area” systems. Additional network segments can be necessary in any of the “Ubiquity level” types of system depending on the specific functionalities of the concrete application. An example of this is the study case of the present paper. In the LOBIN [44] system a WAN/Backhaul segment is added in order to allow for remote access to the data base through the Internet.

A study on the communications technologies that can be used in each type of network segment has also been performed. The following discussion analyzes how traffic density and delivery frequency system properties make some technologies more appropriate than others.

- Projects which sources generate little traffic, like Nerino *et al.* [50] and Zhang *et al.* [49] or low delivery frequencies (order of seconds or minutes), can afford the use of technologies providing lower bandwidth that allow for lesser energy consumption in the BAN and PAN network segments, without incurring in packet losses. Such technologies are MICS, ANT, IEEE 802.15.4/Zigbee and some proprietary communication technologies in the ISM band. Despite this, some other projects with low traffic pattern use more popular technologies that involve higher power consumptions, such as Wi-Fi (IEEE 802.11) and Bluetooth, for monetary or technology compatibility reasons.
- Projects with information sources generating a higher amount of traffic, such as AUBADE [31] or LifeGuard [22], or with higher delivery frequencies, like Mithril LiveNet [17,18] (order of miliseconds) require communication technologies with a higher bandwidth and therefore higher energy consumption in the BAN or PAN/AN network segments. The communication technologies most commonly used in such systems are Bluetooth, Wi-Fi (IEEE 802.11) and wired technologies.
- There are also systems, like LOBIN [44], CodeBlue [25] and MASN [38] that, despite handling a high level of traffic, rely in IEEE 802.15.4/Zigbee in order to optimize energy consumption and decrease the size of the batteries and therefore the size of the wearable devices. These projects assume the loss of a small percentage of packets and compensate the bandwidth restrictions of the IEEE 802.15.4 technology with a careful design of several communication protocols.

Depending on the number of monitored patients some network design decisions that can dramatically improve the overall system performance:

- In “Controlled Area” systems with simultaneous monitoring of multiple patients, such as LOBIN [44], MASN [38] and MEDISN [46], typically a single wireless traffic source is used to monitor each patient in order to minimize the physical medium occupancy. In case multiple sensors are used to monitor each patient, it is common to use a **“**concentrator of measurements**”** (through a BAN) and send them all at once. These projects often introduce an AN network segment (instead of a PAN) in the communications architecture in order to properly distribute the traffic and avoid bottlenecks, providing scalability to the WHMS. In such AN segment of the studied projects different topologies can be found, namely a network of relay points as in MASN [38], a multiple gateway network as in LOBIN [44], or a mesh network as in MEDISN [46].
- In “Controlled Area” systems in which the network is not shared by several patients as Lee *et al.* [42], or in “Wide Area” and “Self-Monitoring” systems in which the patients have their own networks (as in AMON [19] and HealthGear [28] respectively), there are no problems of scalability and distribution of traffic.

## 5. Conclusions

This paper presents a comprehensive review and update on architectures and communications technologies for wearable healthcare systems from a very practical perspective. A general communications architecture for this kind of systems is sketched out and the most appropriate communications technologies are analyzed, depending on the specific requirements of each communications segment of such architecture. The most relevant projects in the area are also summarized in easy-handling format, what facilitates their comparison, and detailed in chronological order so that the reader can easily get an impression of the evolution and current trends of WHMS. In addition, the paper details the design, development, and validation phases of a real-world hardware and software IT platform (the LOBIN platform), based on e-textile and WSN as most innovative technologies, to monitor a set of physiological parameters from a group of patients and to locate them (or any other personnel from the hospital if desired) within hospital facilities. Finally, the most appropriate design criteria and decision making depending on the specific requirements of the target applications are discussed.

## Figures and Tables

**Table 1.** Summary of Communications Technologies for WBAN.

	MICS	IEEE 802.15.6	ANT	Zigbee	Bluetooth 4.0 (LE)

Radio & MAC Stds	FCC and ETSI Stds	IEEE 802.15.6	Propr.	IEEE 802.15.4	IEEE 802.15.1
Frequency Band	402–405 MHz	NB: 402–405/420–450/863–870/902–928/950–956/2,360–2,400/2,400–2,483.5 MHzUWB: 3–10 GHzHBC: 16/27 MHz	2,4–2,524 GHz: 125 1 MHz channels	868 MHz/915 MHz/2.4 GHz	2.4 GHz
Data Rate	Up to 500 Kbps	NB: 57.5–485.7 KbpsUWB: 0.5–10 Mbps		20–250 Kbps	50–200 Kbps
Range	2 m	1.2 m	Up to 10 m	30 m	30 m
Tx Power	25 μW	0.1 μW	0.01–1 mW	30 mW	∼10 mW
Network Topology			Star, tree, mesh	Star, tree, mesh	Star
Stack size			16 KB	100 KB	250 KB

**Table 2.** Comparison of selected WHMS.

**Project Name**	**Medical Area**	**Ubiquity Level**	**Network Segments**		

			BAN	PAN/AN	WAN/Backhaul

MIThril LiveNet [2003] [17,18]	Behavior	Controlled Area	Wd	WF	
AMON [2004] [19]	General Purpose	Wide Area	Wd		C
Fensli *et al.* [2005] [20]	Cardiac	Wide Area	Ws		C
RECAD [2005] [21]	Cardiac	Wide Area	Ws	*	*
LifeGuard [2005] [22]	General purpose	Self-Monitoring	Wd	B	
MagIC [2005] [23]	Cardiac	Controlled Area	B		
WEALTHY [2005] [24]	Cardiac	Wide Area	eT		C
CodeBlue [2005] [25]	General purpose	Controlled area	15.4	15.4	
Jovanov *et al.* [2005–2006] [26,27]	General purpose	Wide Area and self-monitoring	Z	WF,B, U	C
HealthGear [2006] [28]	Sleep	Self-monitoring	B		
MERMOTH [2006] [29]	General purpose	Wide Area and self-monitoring	eT	WF	I
MyHeart [2006] [30]	Cardiac	Wide Area and self-monitoring	Wd	B	C
AUBADE [2006] [31]	Behavior	Controlled area	Wd	WF,B	
C. Park *et al.* [2006] [32]	Cardiac	Controlled area	Ws	WF,Wd, U	
SmartVest [2007] [33]	General purpose	Controlled area	Ws	U	
Human++ [2007] [34]	General purpose	Controlled area	Ws	U	
Personal Health Monitor [2007] [35]	General purpose	Wide Area and self-monitoring	B		C
Chung *et al.* [2008] [36]	Cardiac	Controlled area	15.4		
Chung et al. [2008] [37]	Cardiac	Wide Area	15.4		C
MASN [2008] [38]	Cardiac	Controlled area	Wd	15.4	
HipGuard [2008] [39]	Hip	Wide Area and self-monitoring	A	B, WF	C
HeartToGo [2009] [40]	Cardiac	Self-monitoring	Wd	B	
Chen *et al.* [2009] [41]	Brain	Wide Area	Z		I
Lee *et al.* [2009] [42]	General purpose	Self-monitoring	eT	15.4	
O'Donovan *et al.* [2009] [43]	Falls	Wide Area	Z		C
LOBIN [2010] (Study CASE) [44]	Cardiac	Controlled area	eT	15.4	I
Yuce *et al.* [2010] [45]	General purpose	Controlled area	MICS	WMTS	I
MEDISN [2010] [46]	General purpose	Controlled area	15.4	15.4	I
Shengh *et al.* [2011] [47]	Cardiac	Self-monitoring	B	WF	C
Kim *et al.* [2011] [48]	Posture	Controlled area	Ws		
Zhang *et al.* [2011] [49]	Posture	Controlled area	15.4		
Nerino *et al.* [2011] [50]	Activity	Controlled area	15.4		
Garverick *et al.* [2011] [51]	Cardiac	Wide Area	Wd	B	C
GeM-REM [2011] [52]	Cardiac	Wide Area	Ws		C
Rofouei *et al.* [2011] [53]	Sleep	Wide Area and self-monitoring	Wd	B	*
KNOWME [2012] [54]	Activity	Wide Area	B		C

B: Bluetooth; C: Cellular Network (e.g., GSM, GPRS; UMTS); eT: e-textile or integrated conductive yarns; I: Regular Internet connection (e.g., ADSL, cable); U: USB; Wd: Wired; WF: Wi-Fi; Ws: Wireless proprietary/not specified; Z: Zigbee; 15.4: IEEE 802.15.4; *: See appropriate table.

**Table 3.** Summary of MIThril LiveNet.

Project Name/Funding	MIThril LiveNet [2003] [17,18]/MIT	
Target Application	General purpose individual and distributed group-based context-aware applications platform (mainly healthcare monitoring and social interaction dynamics analysis).	
Tracked Parameters	Physiological	ECG, EMG, GSR, T, (additional commercial sensors can be interfaced to the system)
	Non-physiological	Acc, L, Voice
System Architecture	PDA with GPS and wearable microcontroller board with sensors. Classifier and inter-process communications software.	
Communication Technologies	BAN	Wired
	PAN/AN	Wi-Fi (IEEE 802.11b)
	WAN/Backhaul	
System Highlights	Real time context-aware classification capabilities.	

**Table 4.** Summary of AMON.

Project Name/Funding	AMON [2004] [19]/EU IST FP5	
Target Application	Unobtrusive monitoring of physiological parameters.	
Tracked Parameters	Physiological	BPr, ECG, HR, SpO2, T
	Non- Physiological	Acc
System Architecture	Wrist-worn, on-board GSM transceiver and sensors.	
Communication Technologies	BAN	Wired
	PAN/AN	
	WAN/Backhaul	Cellular network (GSM)
System Highlights	Sending of sensed data three times a day or on demand. Sending of alerts and sensing data values if any parameter goes out of range.	

**Table 5.** Summary of Fensli *et al.*

Project Name/Funding	Fensli *et al.* [2005] [20]/Norwegian Research Council (MEDKAP project)	
Target Application	Real time and remote heart monitoring.	
Tracked Parameters	Physiological	ECG
	Non- Physiological	
System Architecture	Wireless ECG-sensor, PDA and remote web server.	
Communication Technologies	BAN	RF-transmitter at 869.700 MHz
	PAN/AN	
	WAN/Backhaul	Cellular network (GPRS)
System Highlights	Real time ECG monitoring and report to physicians of ECG alarm situations. Web based diagnosis feedback to patient.	

**Table 6.** Summary of RECAD.

Project Name/Funding	RECAD [2005] [21]/University of Blaise Pascal Clermont-Ferrand	
Target Application	Real time remote and continuous cardiac arrhythmias detection and monitoring.	
Tracked Parameters	Physiological	ECG
	Non- Physiological	
System Architecture	Wireless ECG-sensor, PDA and remote web server.	
Communication Technologies	BAN	Wireless (not specified)
	PAN/AN	Supports Wi-Fi, Bluetooth or digital radio communication
	WAN/Backhaul	Supports wired network mediums (classical modem, ADSL, ISDN, Ethernet, *etc.*) and wide wireless technologies (GSM, GPRS, UMTS and satellite)
System Highlights	Alarm message is generation in case of cardiac abnormal event.	

**Table 7.** Summary of LifeGuard.

Project Name/Funding	LifeGuard [2005] [22]/Stanford University and NASA	
Target Application	Monitoring in extreme situations.	
Tracked Parameters	Physiological	BPr, ECG, HR, R, Sp02
	Non- Physiological	P
System Architecture	Sensors connected by cables to a custom device.	
Communication Technologies	BAN	Serial cable
	PAN/AN	Bluetooth
	WAN/Backhaul	
System Highlights	Monitoring data can be digitally stored during 9 hours on the device or streamed to a base station.	

**Table 8.** Summary of MagIC.

Project Name/Funding	MagIC [2005] [23]/University of Milan, Bioeng. Centre & Cardiac Rehab. Unit	
Target Application	Unobtrusive recording of cardiac patients in clinical environment.	
Tracked Parameters	Physiological	ECG, R
	Non- Physiological	Acc
System Architecture	Vest with sensors and portable electronic board.	
Communication Technologies	BAN	Bluetooth
	PAN/AN	
	WAN/Backhaul	
System Highlights	Transmission of tracked signals to a remote computer or PDA for data storage.	

**Table 9.** Summary of WEALTHY.

Project Name/Funding	WEALTHY [2005] [24]/EU IST FP5	
Target Application	Cardiovascular diseases monitoring during rehabilitation phase.	
Tracked Parameters	Physiological	ECG, R, T
	Non- Physiological	A
System Architecture	Sensors, electrodes and connections integrated in fabric form.	
Communication Technologies	BAN	e-textile
	PAN/AN	
	WAN/Backhaul	GPRS
System Highlights	Sending of monitoring signals in quasi-real time.	

**Table 10.** Summary of CodeBlue.

Project Name/Funding	CodeBlue [2005] [25]/Harvard University	
Target Application	Real time monitoring of multiple patients in hospitals or clinics.	
Tracked Parameters	Physiological	ECG, EMG, HR, SpO2
	Non- Physiological	L
System Architecture	Wrist strap, finger sensors and EMG sensors.	
Communication Technologies	BAN	IEEE 802.15.4
	PAN/AN	IEEE 802.15.4
	WAN/Backhaul	
System Highlights	Monitoring of multiple patients.	

**Table 11.** Summary of Jovanov *et al.*

Project Name/Funding	Jovanov *et al.* [2005–2006] [26,27]/University of Alabama	
Target Application	Ambulatory monitoring during normal daily activities.	
Tracked Parameters	Physiological	ECG, SpO2
	Non- Physiological	Humidity of the environment, L (GPS), M, P, T
System Architecture	Tier 1: WBAN (based on TelosB hardware); Tier 2: Personal server (PDA or Phone); Tier 3: Medical Services.	
Communication Technologies	BAN	Zigbee
	PAN/AN	USB, Wi-Fi, Bluetooth
	WAN/Backhaul	Cellular network (GPRS)
System Highlights	Real time analysis of sensing data with feedback to the user. Generation of warnings based on the user's and environment state.	

**Table 12.** Summary of HealthGear.

Project Name/Funding	HealthGear [2006] [28]/Microsoft	
Target Application	Real time monitoring, visualization and analysis of physiological signals while sleeping.	
Tracked Parameters	Physiological	HR, SpO2
	Non- Physiological	
System Architecture	Wireless sensors connected to a mobile phone via Bluetooth.	
Communication Technologies	BAN	Bluetooth
	PAN/AN	
	WAN/Backhaul	
System Highlights	Real time monitoring and transmission to a cell phone which analyses and shows the physiological signals.	

**Table 13.** Summary of MERMOTH.

Project Name/Funding	MERMOTH [2006] [29]/EU IST FP6	
Target Application	Medical monitoring system.	
Tracked Parameters	Physiological	ECG, R, T
	Non- Physiological	A, P
System Architecture	Garment with sensors, a microcontroller, a PDA with transceiver and a remote PC.	
Communication Technologies	BAN	e-textile
	PAN/AN	Wi-Fi
	WAN/Backhaul	Internet connection
System Highlights	Real time feedback to the patient and remote processing and display.	

**Table 14.** Summary of MyHeart.

Project Name/Funding	MyHeart [2006] [30]/EU IST FP6	
Target Application	ECG monitoring and classification of continuous motion activity.	
Tracked Parameters	Physiological	ECG, HR
	Non- Physiological	A
System Architecture	T-shirt with sensors and processing electronics module, and mobile phone.	
Communication Technologies	BAN	Wired
	PAN/AN	Bluetooth
	WAN/Backhaul	Cellular network (GSM)
System Highlights	Data storing capabilities included in the on-body electronics. Java program for user interaction in mobile phone.	

**Table 15.** Summary of AUBADE.

Project Name/Funding	AUBADE [2006] [31]/EU IST FP5	
Target Application	Emotional assessment of individuals under stress conditions (car racing drivers or patients suffering from neurological and psychological disorders).	
Tracked Parameters	Physiological	ECG, Facial EMG, GSR, R
	Non- Physiological	
System Architecture	A multi-sensorial wearable, composed of three pieces, a data acquisition and wireless communication module, and a centralized system for analysis.	
Communication Technologies	BAN	Wired
	PAN/AN	Bluetooth, Wi-Fi (IEEE 802.11b)
	WAN/Backhaul	
System Highlights	Real-time emotional assessment and data storage.	

**Table 16.** Summary of C. Park *et al.*

Project Name/Funding	C. Park *et al.* [2006] [32]/NSF-CAREER, Army and Air Force of USA	
Target Application	ECG remote monitoring.	
Tracked Parameters	Physiological	ECG
	Non- Physiological	
System Architecture	ECG sensors, base station and computer.	
Communication Technologies	BAN	1 Mbps proprietary radio
	PAN/AN	USB, Ethernet (IEEE 802.3), Wi-Fi (IEEE 802.11 b/g)
	WAN/Backhaul	
System Highlights	Base station sends the information to whichever device connected to it.	

**Table 17.** Summary of SmartVest.

Project Name/Funding	SmartVest [2007] [33]/National Programme on Smart Materials (NPSM), India	
Target Application	Monitoring of the health status and geo-location of employees to ensure the safety and the effective completion assigned tasks.	
Tracked Parameters	Physiological	BPr, ECG, GSR, HR, Photoplethysmogram, T
	Non- Physiological	L (GPS)
System Architecture	Vest with e-textile sensors integrated, wearable data acquisition and processing hardware and remote monitoring station.	
Communication Technologies	BAN	Proprietary from Xtream in the ISM band (2.4 GHz)
	PAN/AN	USB
	WAN/Backhaul	
System Highlights	Continuous transmission of data to remote monitoring station.	

**Table 18.** Summary of Human++.

Project Name/Funding	Human++ [2007] [34]/IMEC-Nl/Holst Centre, Eindhoven, The Netherlands	
Target Application	Ambulatory multi-parameter monitoring.	
Tracked Parameters	Physiological	ECG, EEG, EMG
	Non- Physiological	
System Architecture	Sensors, base station and PDA or PC.	
Communication Technologies	BAN	RF 2.4 GHz ISM
	PAN/AN	USB
	WAN/Backhaul	
System Highlights	Acquisition, processing, storing and visualization of physiological parameters in an unobtrusive way.	

**Table 19.** Summary of Personal Health Monitor.

Project Name/Funding	Personal Health Monitor [2007] [35]/Microsoft	
Target Application	Personal health monitoring.	
Tracked Parameters	Physiological	BPr, ECG, SpO2, Weight
	Non- Physiological	P, L
System Architecture	Wireless sensors and mobile phone with specific application.	
Communication Technologies	BAN	Bluetooth
	PAN/AN	
	WAN/Backhaul	Cellular network
System Highlights	The mobile phone application analyses, in real-time, data wirelessly received from the sensors, and can send this data in real-time to medical specialists. If any parameter goes out of the normal range, the mobile phone automatically determines the person's current location and generates an alert message.	

**Table 20.** Summary of Chung *et al.*

Project Name/Funding	Chung *et al.* [2008] [36]/Ministry of Commerce, Industry and Energy (MOCIE) and Korea Industrial Technology Foundation(KOTEF)	
Target Application	Ubiquitous monitoring system.	
Tracked Parameters	Physiological	ECG, SpO2
	Non- Physiological	A
System Architecture	Chest belt, wrist bandage and monitoring system (PC).	
Communication Technologies	BAN	IEEE 802.15.4
	PAN/AN	
	WAN/Backhaul	
System Highlights	Real-time monitoring and data analysis.	

**Table 21.** Summary of Chung *et al.*

Project Name/Funding	Chung *et al.* [2008] [37]/Consortium research foundation between industry, university and institute by Small and Business Administration in 2006, Korea	
Target Application	Ubiquitous monitoring system.	
Tracked Parameters	Physiological	BPr, ECG
	Non- Physiological	
System Architecture	Chest belt, wrist bandage, mobile phone and remote monitoring system (PC).	
Communication Technologies	BAN	IEEE 802.15.4
	PAN/AN	
	WAN/Backhaul	Cellular network
System Highlights	Real-time and local monitoring and data analysis. Remote transmission of data in case of suspicious pattern detection.	

**Table 22.** Summary of MASN.

Project Name/Funding	MASN [2008] [38]/University of Alabama	
Target Application	Remote cardiac monitoring of multiple patients.	
Tracked Parameters	Physiological	ECG
	Non- Physiological	L (indoor)
System Architecture	ECG micro-sensor with RF motes and ECG server.	
Communication Technologies	BAN	Wired
	PAN/AN	IEEE 802.15.4
	WAN/Backhaul	
System Highlights	Real time data collection and data mining. Multiple patients monitoring in a mesh architecture.	

**Table 23.** Summary of HipGuard.

Project Name/Funding	HipGuard [2008] [39]/Tampere University of technology, Finland	
Target Application	Assessment of patients recovering from hip surgery.	
Tracked Parameters	Physiological	
	Non- Physiological	P and rotation (of leg and hip). Force between foot and a shoe.
System Architecture	3-axis accelerometers, 3-axis magnetometers, 1 Capacity insole sensor, wrist unit and a mobile phone.	
Communication Technologies	BAN	ANT
	PAN/AN	Bluetooth, Wi-Fi
	WAN/Backhaul	Cellular network (GPRS, 3G)
System Highlights	Sending of signals to remote server for remote monitoring. Sending of alarm signals to patient's Wrist Unit.	

**Table 24.** Summary of HeartToGo.

Project Name/Funding	HeartToGo [2009] [40]/University of Pittsburgh	
Target Application	Real-time monitoring and recording of ECG.	
Tracked Parameters	Physiological	ECG
	Non- Physiological	L
System Architecture	ECG sensor, GPS receiver and mobile phone with specific application.	
Communication Technologies	BAN	Wired
	PAN/AN	Bluetooth
	WAN/Backhaul	
System Highlights	Real time monitoring and recording, feature extraction, report generation and health assessment.	

**Table 25.** Summary of Chen *et al.*

Project Name/Funding	Chen *et al.* [2009] [41]/Korea Research Foundation Grant funded by Korea Government	
Target Application	Monitoring of brain bioelectrical activity.	
Tracked Parameters	Physiological	EEG
	Non- Physiological	
System Architecture	Composed of Zigbee EEG sensors and a Zigbee/Internet Gateway (ZiGW).	
Communication Technologies	BAN	Zigbee
	PAN/AN	
	WAN/Backhaul	Internet
System Highlights	EEG data is stored in online database in real time.	

**Table 26.** Summary of Lee *et al.*

Project Name/Funding	Lee *et al.* [2009] [42]/Dongseo University, Korea	
Target Application	Battlefield, public safety, health monitoring, sports and fitness, among others.	
Tracked Parameters	Physiological	ECG
	Non- Physiological	Acc
System Architecture	E-textile t-shirt with sensors and PC.	
Communication Technologies	BAN	E-textile
	PAN/AN	IEEE 802.15.4
	WAN/Backhaul	
System Highlights	Continuously collecting data. Use of the acceleration information to enhance ECG signal.	

**Table 27.** Summary of O**'**Donovan *et al.*

Project Name/Funding	O'Donovan *et al.* [2009] [43]/EU FP7	
Target Application	Falls assessment of elder patients.	
Tracked Parameters	Physiological	BPr, ECG, HR, SpO2
	Non- Physiological	M, Acc
System Architecture	BAN of Sensors and a GSM gateway.	
Communication Technologies	BAN	Zigbee
	PAN/AN	
	WAN/Backhaul	Cellular network (GSM)
System Highlights	Continuous acquisition of ECG and BPR of a single patient.	

**Table 28.** Summary of LOBIN.

Project Name/Funding	LOBIN [2010] (Study case) [44]/Spanish Ministry of Industry, Tourism and Trade	
Target Application	Non-invasive and pervasive monitoring of patients with cardiac issues in future healthcare environments.	
Tracked Parameters	Physiological	ECG, HR, T
	Non- Physiological	A, L (indoor)
System Architecture	E-textile t-shirts, location devices, a wireless sensor networks and base stations that forward the data, and a server (see study case section)	
Communication Technologies	BAN	e-textile
	PAN/AN	IEEE 802.15.4
	WAN/Backhaul	Internet
System Highlights	Sending of data either every 260 ms or on-demand, and storage in a server from where data can be accessed in real-time. Alert generation whenever tracked parameters go out of customizable range.	

**Table 29.** Summary of Yuce *et al.*

Project Name/Funding	Yuce *et al.* [2010] [45]/Australian Research Council (ARC) under Discovery Projects Grant	
Target Application	Monitoring of physiological signals from patients in medical environments.	
Tracked Parameters	Physiological	BPr, ECG, EEG, EMG, GSR, R, T
	Non- Physiological	
System Architecture	Sensors, a multi-hop WBAN with a receiving device and a local or remote PC.	
Communication Technologies	BAN	MICS
	PAN/AN	WMTS
	WAN/Backhaul	Internet
System Highlights	Data from sensors is collected, interpreted and stored in the PC for further analysis by health professionals.	

**Table 30.** Summary of MEDISN.

Project Name/Funding	MEDISN [2010] [46]/National Science Foundation and Department of Homeland Security, USA	
Target Application	Medical emergency detection in hospital environments or disaster events.	
Tracked Parameters	Physiological	ECG, HR, SpO2
	Non- Physiological	
System Architecture	RF sensors with built-in display, relay points, a gateway and a remote database.	
Communication Technologies	BAN	IEEE 802.15.4
	PAN/AN	IEEE 802.15.4
	WAN/Backhaul	Internet
System Highlights	Periodical data sending through a WSN mesh and data storing in a server.	

**Table 31.** Summary of Shengh *et al.*

Project Name/Funding	Shengh *et al.* [2011] [47]/Michigan Technological University, Houghton, USA	
Target Application	ECG monitoring for cardiac arrhythmia classification.	
Tracked Parameters	Physiological	ECG
	Non- Physiological	A
System Architecture	Wearable ECG sensor node and mobile phone	
Communication Technologies	BAN	Bluetooth
	PAN/AN	WiFi
	WAN/Backhaul	GPRS
System Highlights	Real-time analysis and diagnosis of cardiovascular diseases.	

**Table 32.** Summary of Kim *et al.*

Project Name/Funding	Kim *et al.* [2011] [48]/University of Virginia Biomedical Innovation Fund and the National Science Foundation under grants CBET	
Target Application	Combat of “Forward Head Posture” and reduction of the associated neck pain.	
Tracked Parameters	Physiological	
	Non- Physiological	Cranium-vertebral angle (CV), P
System Architecture	Body-mounted accelerometers and a laptop.	
Communication Technologies	BAN	Wireless not specified
	PAN/AN	
	WAN/Backhaul	
System Highlights	CV angle is assessed and compared with FHP thresholds. A beep sounds for auto-correction.	

**Table 33.** Summary of Zhang *et al.*

Project Name/Funding	Zhang *et al.* [2011] [49]/Imperial College London, UK	
Target Application	Assessment of patients with back pain.	
Tracked Parameters	Physiological	
	Non- Physiological	M (human back)
System Architecture	3 axis accelerometer, a gyroscope, a magnetometer and a base station connected to a PC.	
Communication Technologies	BAN	IEEE 802.15.4
	PAN/AN	
	WAN/Backhaul	
System Highlights	Euler angles are defined to represent the orientation for human back segments. Kinematics analysis is then derived.	

**Table 34.** Summary of Nerino *et al.*

Project Name/Funding	Nerino *et al.* [2011] [50]/EU FP7	
Target Application	Assessment of the hippo-therapy.	
Tracked Parameters	Physiological	
	Non- Physiological	M (of rider and horse)
System Architecture	Two 3-axis accelerometers sensors, an IEEE 802.15.4 gateway connected to a laptop and a webcam.	
Communication Technologies	BAN	IEEE 802.15.4
	PAN/AN	
	WAN/Backhaul	
System Highlights	A Matlab script carries out a cross-correlation analysis of accelerations measured on the rider-horse.	

**Table 35.** Summary of Garverick *et al.*

Project Name/Funding	Garverick *et al.* [2011] [51]/West Wireless Health Institute	
Target Application	Cardiotocography	
Tracked Parameters	Physiological	HR (fetal)
	Non- Physiological	Uterine contractions
System Architecture	Belt with toco sensor, fetal heart rate monitor device and optionally a cellular gateway.	
Communication Technologies	BAN	Wired
	PAN/AN	Bluetooth
	WAN/Backhaul	Cellular network
System Highlights	Built-in 4.5 hours monitoring back-up memory.	

**Table 36.** Summary of GeM-REM.

Project Name/Funding	GeM-REM [2011] [52]/National Science Foundation, USA	
Target Application	ECG monitoring.	
Tracked Parameters	Physiological	ECG
	Non- Physiological	
System Architecture	ECG leads, sensor platform, mobile phone gateway, base station (computer).	
Communication Technologies	BAN	Wireless (not specified)
	PAN/AN	
	WAN/Backhaul	Cellular
System Highlights	Resource-efficient (energy consumption and data size at the sensor) ECG monitoring based on a generative ECG model at the base station and its lightweight version at the sensor: the sensor transmits data only when the sensed ECG deviates from model based values and the model parameters are continually updated based on the sensed ECG.	

**Table 37.** Summary of Rofouei *et al.*

Project Name/Funding	Rofouei *et al.* [2011] [53]/UCLA, Microsoft Research	
Target Application	Real-time sleep monitoring and pre-diagnosis of sleep apnea.	
Tracked Parameters	Physiological	R, SpO2
	Non- Physiological	P (of the head)
System Architecture	Neck-cuff (with sensor nodes, microprocessor and Bluetooth transceiver) and cell phone or desktop machine.	
Communication Technologies	BAN	Wired
	PAN/AN	Bluetooth
	WAN/Backhaul	Cloud (from mobile or laptop)
System Highlights	Real time data acquisition and visualization, and cloud aggregation.	

**Table 38.** Summary of KNOWME.

Project Name/Funding	KNOWME [2012] [54]/University of Southern California, USA	
Target Application	Monitoring and evaluation of physical activity of pediatric obesity patients.	
Tracked Parameters	Physiological	ECG, SpO2
	Non- Physiological	L(GPS), A, Acc
System Architecture	WBAN of sensors and a mobile phone (Nokia N95).	
Communication Technologies	BAN	Bluetooth
	PAN/AN	
	WAN/Backhaul	Cellular network (GPRS/UMTS)
System Highlights	Continuous long term data collection to determine energy expenditure of patients. Inference of physical state.	

**Table 39.** Healthcare Monitoring Subsystem Parameters.

**Parameter**	**Features**
ECG	Frequency: 0.5–125 Hz Amplitude: ∼0–50 mV Gain: G 1 byte/sample 65 samples/tx packet
Heart rate	Computed from ECG 1 byte/sample 1 sample/tx packet
Angle of inclination	Computed from 3-axis accelerometer 1 byte/sample 1 sample/tx packet
Activity index	Computed by averaging angle of inclination 1 byte/sample 1 sample/tx packet
Body temperature	Range: −20 °C to 120 °C Accuracy: 0.1 °C Valid range: 32 °C to 45 °C 1 byte/sample 1 sample/tx packet
Level of battery	100–0% Coverage: 8–9 hours 1 byte/sample 1 sample/tx packet
Alert	To carry alert code if necessary 1 byte/sample 1 sample/tx packet

**Table 40.** Location Subsystem Tested Configurations.

**Test**	**Ptx_loc (s)**	**Tlisten (s)**	**Pbeacons (s)**

1	30	2	1
2	9	2	1
3	20	4	1
4	15	2	0.5
5	9	2	0.5

## Acronyms

A: Activity
Acc: Acceleration
AGPS: Assisted-Global Position System
AMON: Care and Alert portable telemedical MONitor
BAN: Body Area Network
BP: Beacon Point
BPr: Blood Pressure
DAPB: Data Acquisition and Processing Board
DP: Distribution Point
DSR: Dynamic Source Routing
E-OTD: Enhanced Observed Time Difference
ECG: Electrocardiogram
EEG: Electroencephalogram
EMG: Electromyogram
ETB: Ethernet Transmission Board
GeM-REM: Generative Model-driven Resource efficient ECG Monitoring in Body Sensor Networks
GPRS: General Packet Radio Service
GSM: Global System for Mobile Communication
GSR: Galvanic Skin Response
GUI: Graphical User Interface
HR: Heart Rate
IT: Information Technology
L: Location
LAN: Local Area Network
LBS: Location Based Services
LOBIN: *LOcalización y Biomonitorización de pacientes a través de redes INalámbricas en entornos hospitalarios*
LQI: Link Quality Indicator
M: Movement
MAC: Media Access Control
MAGIC: *Maglietta Interattiva Computerizzata*
MASN: Medical Ad hoc Sensor Networks
MEDIsN: Medical Emergency Detection In Sensor Networks
MERMOTH: MEdical Remote MOnitoring with cloTHes
MICS: Medical Implant Communications Service
MTU: Maximum Transfer Unit
Nchannels: Number of channels available for sending location beacons
P: Position
PAN: Personal Area Network
Pbeacons: Period Beacon Points send beacons with
PDA: Personal Digital Assistant
PCB: Printed-Circuit Board
Ptx_loc: Period of time LWTBs send location information with
R: Respiration
RECAD: REal-time Continuous Arrhythmias Detection system
RFID: Radio Frequency Identification
RSSI: Received Signal Strength Indicator
SMS: Short Message Service
SpO2: Blood Oxygen Saturation
T: Temperature
TDoA: Time Difference of Arrival
Tlisten: Time LWTBs spend listening to each channel available for location
ToA: Time of Arrival
UMTS: Universal Mobile Telecommunications System
UWB: Ultra Wide Band
WAN: Wide Area Network
WDAD: Wearable Data Acquisition Device
WHMS: Wearable Healthcare Monitoring System
WLAN: Wireless Local Area Network
WMTS: Wireless Medical Telemetry Service
WSN: Wireless Sensor Network
WTB: Wireless Transmission Board
